# Drug Screening Identifies Niclosamide as an Inhibitor of Breast Cancer Stem-Like Cells

**DOI:** 10.1371/journal.pone.0074538

**Published:** 2013-09-18

**Authors:** Yu-Chi Wang, Tai-Kuang Chao, Cheng-Chang Chang, Yi-Te Yo, Mu-Hsien Yu, Hung-Cheng Lai

**Affiliations:** 1 Graduate Institute of Medical Sciences, National Defense Medical Center, Taipei, Taiwan (R.O.C.); 2 Department of Obstetrics and Gynecology, Tri-Service General Hospital, National Defense Medical Center, Taipei, Taiwan (R.O.C.); 3 Laboratory of Epigenetics and Cancer Stem Cells, National Defense Medical Center, Taipei, Taiwan (R.O.C.); 4 Department of Pathology, Tri-Service General Hospital, National Defense Medical Center, Taipei, Taiwan (R.O.C.); Florida International University, United States of America

## Abstract

The primary cause of death from breast cancer is the progressive growth of tumors and resistance to conventional therapies. It is currently believed that recurrent cancer is repopulated according to a recently proposed cancer stem cell hypothesis. New therapeutic strategies that specifically target cancer stem-like cells may represent a new avenue of cancer therapy. We aimed to discover novel compounds that target breast cancer stem-like cells. We used a dye-exclusion method to isolate side population (SP) cancer cells and, subsequently, subjected these SP cells to a sphere formation assay to generate SP spheres (SPS) from breast cancer cell lines. Surface markers, stemness genes, and tumorigenicity were used to test stem properties. We performed a high-throughput drug screening using these SPS. The effects of candidate compounds were assessed *in vitro* and *in vivo*. We successfully generated breast cancer SPS with stem-like properties. These SPS were enriched for CD44^high^ (2.8-fold) and CD24^low^ (4-fold) cells. OCT4 and ABCG2 were overexpressed in SPS. Moreover, SPS grew tumors at a density of 10^3^, whereas an equivalent number of parental cells did not initiate tumor formation. A clinically approved drug, niclosamide, was identified from the LOPAC chemical library of 1,258 compounds. Niclosamide downregulated stem pathways, inhibited the formation of spheroids, and induced apoptosis in breast cancer SPS. Animal studies also confirmed this therapeutic effect. The results of this proof-of-principle study may facilitate the development of new breast cancer therapies in the near future. The extension of niclosamide clinical trials is warranted.

## Introduction

Breast cancer is one of the most common cancer in women worldwide and was estimated to affect 230,480 incident patients, with 39,520 deaths, in the USA in 2011 [1]. The mortality rate of breast cancer is high because of disease recurrence, which remains the major therapeutic barrier in this type of cancer. Although chemotherapy or radiotherapy can kill most bulky tumor cells and provide temporary remission, relapse occurs in most cases, possibly as a result of the recently proposed cancer stem cell hypothesis [2]. Most research on human cancer has focused on the analysis of the bulky tumor mass. Growing evidence shows that tumor populations are heterogeneous regarding proliferation and differentiation, which raises the concept of “cancer stem cell” and may have profound implications for breast cancer therapy [3].

The cancer stem cell hypothesis implies that a subset of tumor cells has the ability to self-renew and is the source of tumor initiation, progression, and recurrence [3]–[5]. These cancer stem cells may also contribute to tumor formation, metastasis, and treatment resistance [6], [7]. Although the origin and biology of cancer stem cells remain controversial, research in this field is anticipated to provide new approaches to the treatment of cancer. The idea of targeting cancer stem cells using novel compounds that may overcome current chemotherapy and radiation therapy is appealing. In fact, some studies that adopted this concept and approach have successfully identified novel compounds. Studies have shown that some agents (such as metformin) can selectively target cancer stem cells and that dietary polyphenols, curcumin, peperine, and sulforaphane, which are derived from broccoli/broccoli sprouts, are able to target breast cancer stem cells via inhibition of the Wnt signaling, which affects mammosphere size and colony formation [8]–[10]. These studies indicated that strategies that use spheroids as a screening method may be an effective approach to the identification of new compounds that target cancer stem-like cells.

Cancer stem-like cells reside among cancer cell populations; their isolation is complex and remains a challenge. Many methods have been applied to identify breast cancer stem-like cells, including cell-surface markers [11], [12], dye-exclusion side population (SP) cells, sphere formation, and the expression of aldehyde dehydrogenase (ALDH) [13]–[16]. Accumulated evidence has revealed that even “cancer stem cells” are heterogeneous while distinct (sub)populations can be isolated/enriched by different approaches [11], [14], [17], [18]. Although each method has been used and studied individually, they have not been used together to determine the overall profile of cancer stem-like cells. It is logical to combine different methods for the enrichment of cancer stem cells, as this may represent cancer cells at a higher level of cancer hierarchy and be more suitable for drug development.

To circumvent this problem, here we adopted SP cells, followed by the spheroid culture technique, to enrich cancer stem-like cells. We performed a chemical screening using a compound library for the repurposing of old drugs for breast cancer treatment.

## Methods

### Reagents

The LOPAC chemical library containing 1,258 compounds (off-patent drugs and chemical compounds), niclosamide powder (for follow-up experiments), and Hoechst 33342 were purchased from Sigma-Aldrich (St. Louis, MI, USA). Monoclonal antibodies against each of human CD24 and CD44 were purchased from Abcam (Cambridge, UK).

### Cell Culture

The human breast cancer cell lines MCF7 and MDA-MB-231 were obtained from ATCC. Cells were cultured in DMEM supplied with 1% penicillin/streptomycin and 10% fetal bovine serum (FBS; Invitrogen-Life Technologies). All cell lines were incubated in a humidified incubator at 37°C supplied with 5% carbon dioxide.

### Fluorescence-activated Cell Sorting and Side Population Cells

Side population cells were isolated from human MCF7 breast cancer cells using a Hoechst 33342 staining strategy modified as described previously [19]. Briefly, cells were suspended in medium (100,000 cells/mL) and allowed to recover at 37°C for 1 h before Hoechst 33342 staining. The Hoechst dye was added at a concentration of 7 µg/mL and cells were incubated at 37°C for 1 h. The ABCG2 inhibitor GF120918 was added at a final concentration of 50 µg/mL to confirm the gating area on flow cytometry. After incubation, cells were washed and resuspended in HBSS containing 5% FBS. Cells were further stained with 1 µg/mL propidium iodide (Sigma-Aldrich), to assess viability, and were then analyzed and sorted via flow cytometry using a FACSAria apparatus (Becton Dickinson).

### Spheroid Culture

Cells obtained from FACSAria sorting in the primary culture were plated immediately in ultralow attachment plates (Corning) at a density of 2,000 viable cells/mL in serum-free DMEM/F12 medium containing 5 µg of insulin (Sigma), 0.4% bovine serum albumin (Sigma), 10 ng/mL basic fibroblast growth factor; Invitrogen), and 20 ng/mL human recombinant epidermal growth factor (Invitrogen). Subsequently, SP cells were observed under suspension culture conditions to detect the stem cell phenotype of tumorsphere formation [20], [21]; “spheroid”-forming cells were termed side population spheres (SPS).

### High-throughput Drug Screening Assay

MCF7 SP cells were seeded into 96-well ultralow attachment cluster plates at a density of 6,000 cells per well in 100 µL of serum-free medium (Corning). Cells were then treated with compounds from the LOPAC library (Sigma-Aldrich) at concentrations of 3 and 30 µmol/L for 72 h. We assessed the size of spheroids based on the images captured using a cooled, back-thinned CCD camera and analyzed the images using the Image-Pro Plus 6.0 software (Media Cybernetics, Bethesda, MD, USA), which provides a computational algorithm for the analysis of cellular areas. We set the threshold for image capture at a size of 2,000 pixels.

### Cell Viability Assay

Cells were plated at 1,000 cells per well of a 96-well plate for 1 day and were then treated with chemotherapeutic drugs for 72 h. Subsequently, we evaluated cell viability using the CellTiter-Glo (Promega, Annandale, NSW, Australia) luminescent cell viability assay (ATP assay). Briefly, the ATP reagent (0.01 µM) was added to 100 µL of medium containing cells in each well of a 96-well plate and the intensity of luminescence was measured 10 min after the addition of the reagent. All the *in vitro* experiments were done at least in two independent biological replicates and three technical replicates.

### Flow Cytometry

The expression of stem cell markers in MCF7 SPS was evaluated using a BD FACSCalibur apparatus (Becton Dickinson, San Jose, CA). Antibodies against CD44, CD24, OCT4, NANOG, and ABCG2 were purchased from Abcam (Cambridge, UK). Cells were harvested and incubated with specific antibodies at room temperature for 30 min, followed by incubation with FITC-conjugated secondary antibodies for 20 min. Cells were stained with 1 µg/mL propidium iodide (Sigma-Aldrich), to assess viability, and analyzed on a BD FACSCalibur apparatus. Apoptosis was assessed via staining with annexin V (Abcam) and propidium iodide (Sigma-Aldrich) and was analyzed using flow cytometry (FACSCalibur).

### RNA Extraction and RT–PCR

Total RNA of naive and niclosamide-treated MCF7 SPS was isolated using the Qiagen RNeasy kit (Qiagen; Valencia, CA, USA) according the manufacturer’s protocol. One microgram of total RNA from each sample was subjected to cDNA synthesis using Superscript II reverse transcriptase and random hexamers (Invitrogen). A LightCycler FastStart DNA Master SYBR Green I kit (Roche Applied Science; Indianapolis, IN, USA) was used for the quantification of target gene expression via real-time PCR assays performed using a Real-Time PCR instrument (Roche).

### Xenograft Models

NOD/SCID mice were purchased from National Taiwan University. All procedures were approved by the Laboratory Animal Care and Use Committee of the National Defense Medical Center. For studies of tumor xenografts, equal amounts of MCF7 and MCF7 SPS cells suspended in 100 µL of matrigel were injected subcutaneously into the NOD/SCID mice. To assay the effects of treatment with the compounds identified, female NOD/SCID mice (6 weeks old) were housed under pathogen-free conditions at the animal center of the National Defense Medical Center. Treatment with compounds was initiated 24 h after tumor injection. Animals were administered either vehicle (PBS) or niclosamide (10 kg/mg) intraperitoneally 5 days per week for 8 weeks. The groups of mice were killed after 8 weeks and the fat pads were analyzed for the presence of tumor outgrowth.

### Statistical Analysis

The mean and the standard error of the mean are reported. Data were compared using two-tailed and Student’s *t* tests. Differences were considered significant if *P*<0.05.

## Results

### Enrichment of Breast Cancer Stem-like Cells using Combined Dye-exclusion and Spheroid Methods from Breast Cancer Cell Lines

**Figure 1:**
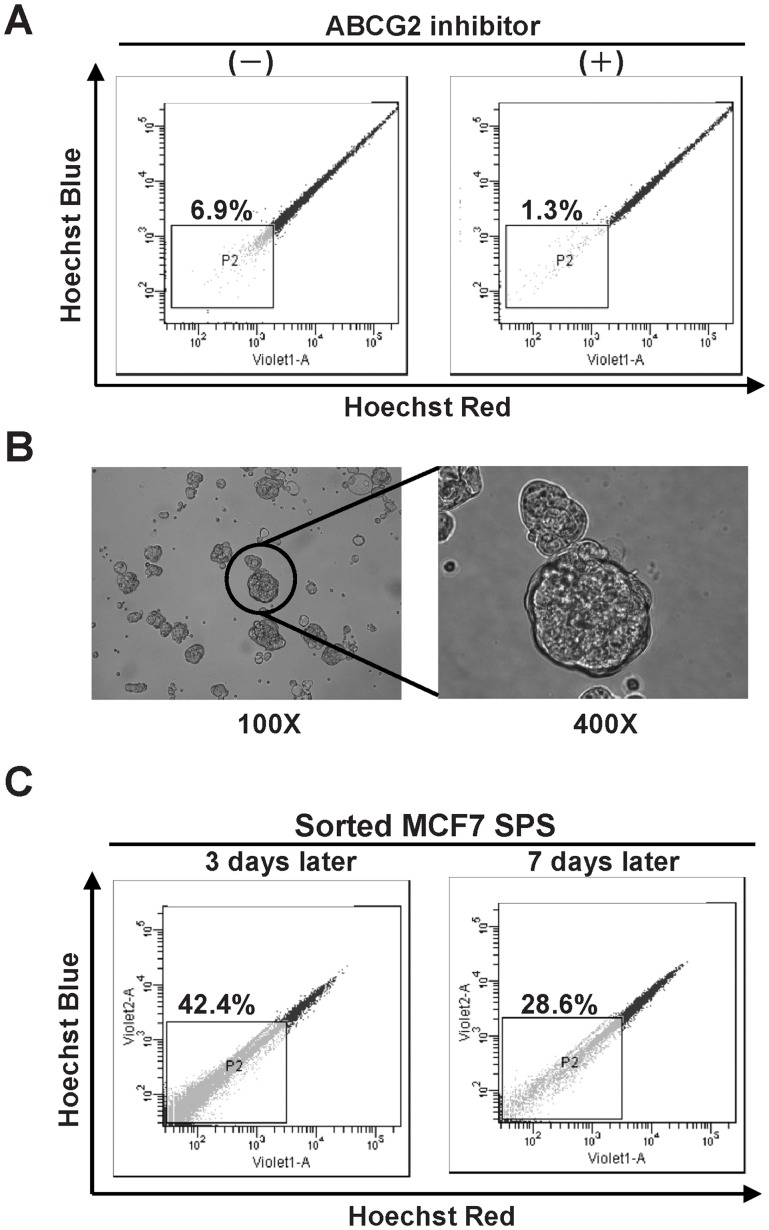
Isolation of breast cancer SPS. (A) MCF7 cell line exhibiting low Hoechst 33342 staining defines the fraction of SP. The presence of the ABCG2 inhibitor GF120918 blocks the exclusion of Hoechest dye and confirms the selection of SP for further studies. (B) MCF7 SPS generated by SP grown in a serum-free suspension culture (magnification, 100× (left) and 400× (right)). (C) MCF7 SPS repopulated both SP and non-SP cells. The fraction of SP decreased during repopulation. (left on Day 3; right on Day 7).

The MCF7 cell line contained SP cells that presented a so-called distinct “tail”, as assessed using flow cytometry [22]. We used Hoechst 33342 to detect and sort these side populations based on their capacity to efflux the fluorescent dye. The SP, representing 6.9% of the total cells, was confirmed using an ABC transporter inhibitor, GP 120918 (Figure 1A). Subsequently, the MCF7 SP was cultured in a serum-free suspension culture for 2 weeks, to grow SPS (Figure 1B). Repopulation of SPS was examined. The SPS were seeded on adherent plates and the SP fraction decreased from 42.4% in day 3 to 28.6% in day 7. This result showed that MCF7 SPS can repopulate both SP and non-SP MCF7 cells (Figure 1C).

**Figure 2:**
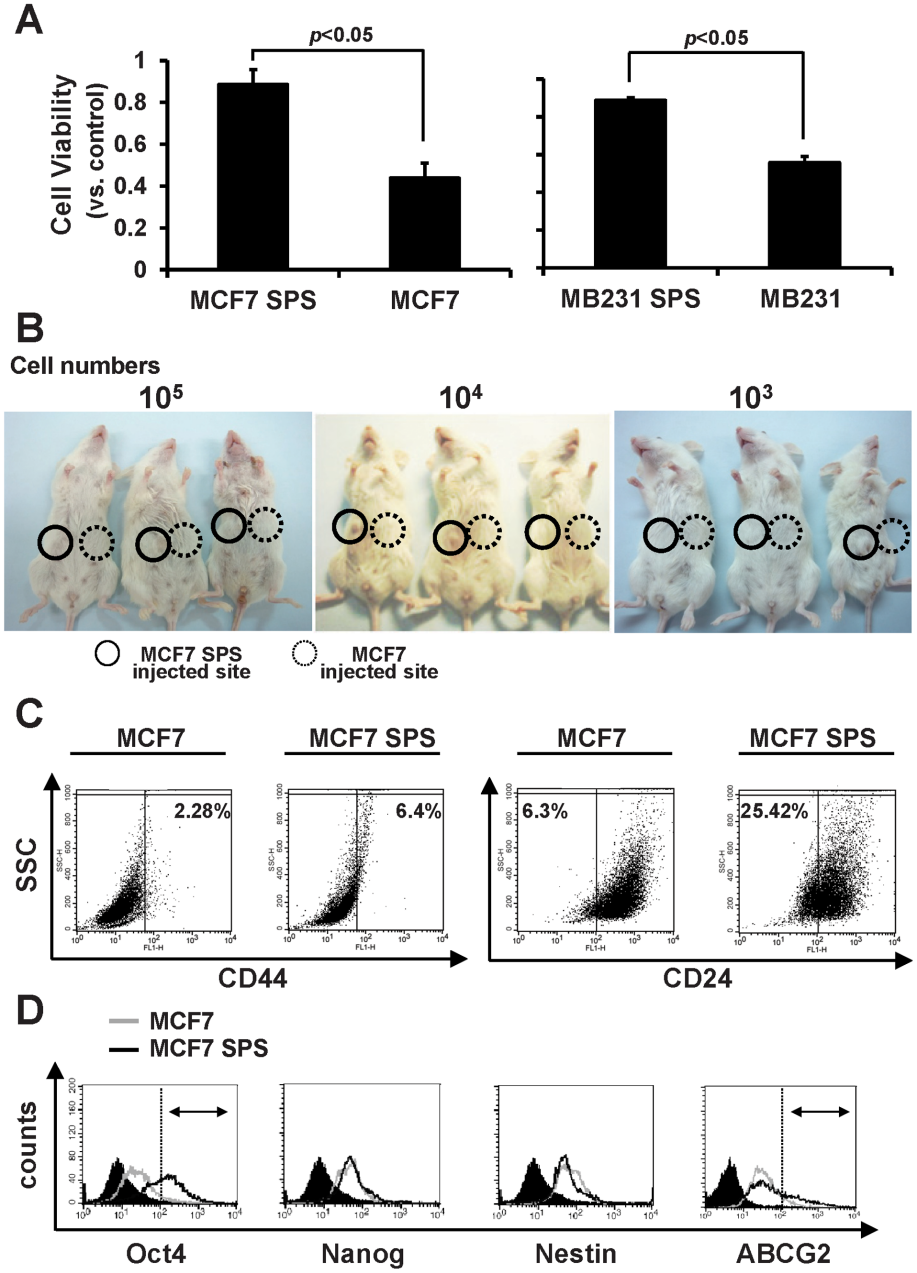
Characterization of Breast cancer SPS. (A) Drug sensitivity of SPS and parental lines to paclitaxol. (B) Tumorigenicity of MCF7 SPS or MCF7 (10^5^, 10^4^ and 10^3^ cells) injected subcutaneously into female NOD/SCID mice. (C) Flow cytometric analysis of CD44 and CD24 on MCF7 SPS. (D) Flow cytometric analysis of stem cell markers, including OCT4, NANOG, Nestin, and ABCG2, in MCF7 SPS. (*P<0.05).

The chemosensitivity of SPS to a commonly used chemotherapeutic agent, paclitaxol, was assessed. Both differentiated and SPS cells of MCF7 and MDA-MB-231 were treated with paclitaxol at 100 nM for 3 days. Cell viability was determined using an ATP assay. As expected, SPS were significantly more resistant to paclitaxol treatment (Figure 2A). To test the cancer stem-like properties of MCF7 SPS, the tumorigenicity of MCF7 SPS and their parental cells was assessed in two independent sets of three NOD/SCID mice. All NOD/SCID mice with MCF7 SPS grew tumors 8 weeks after inoculation, whereas mice with MCF7 cells did not develop tumors at a density of 10^5^ cells. SPS cells exhibited tumorigenic potential at a density as low as 10^3^ cells and no mice injected with an equivalent amount of differentiated cells grew tumors (Figure 2B).

Previously, the CD44^high^CD24^low^ phenotype was reported as breast cancer stem cells [11]. We examined this phenotype in SPS. CD44^high^ cells were enriched in MCF7 SPS by 2.8-fold and CD24^low^ cells were enriched by 4-fold (Figure 2C). This suggests that the marker-free-derived MCF7 SPS and CD44^high^/CD24^low^ detect distinct, but partially overlapping, cancer stem-like cells. Other stem cell markers, including OCT4, NANOG, NESTIN, and ABCG2, were also analyzed using flow cytometry. OCT4 and ABCG2 were enriched in MCF7 SPS (Figure 2D). These data support that MCF7 SPS cells are enriched for stem-like properties.

### Identification of Small Molecules that Inhibit Tumorsphere Formation using High-throughput Screening

**Figure 3:**
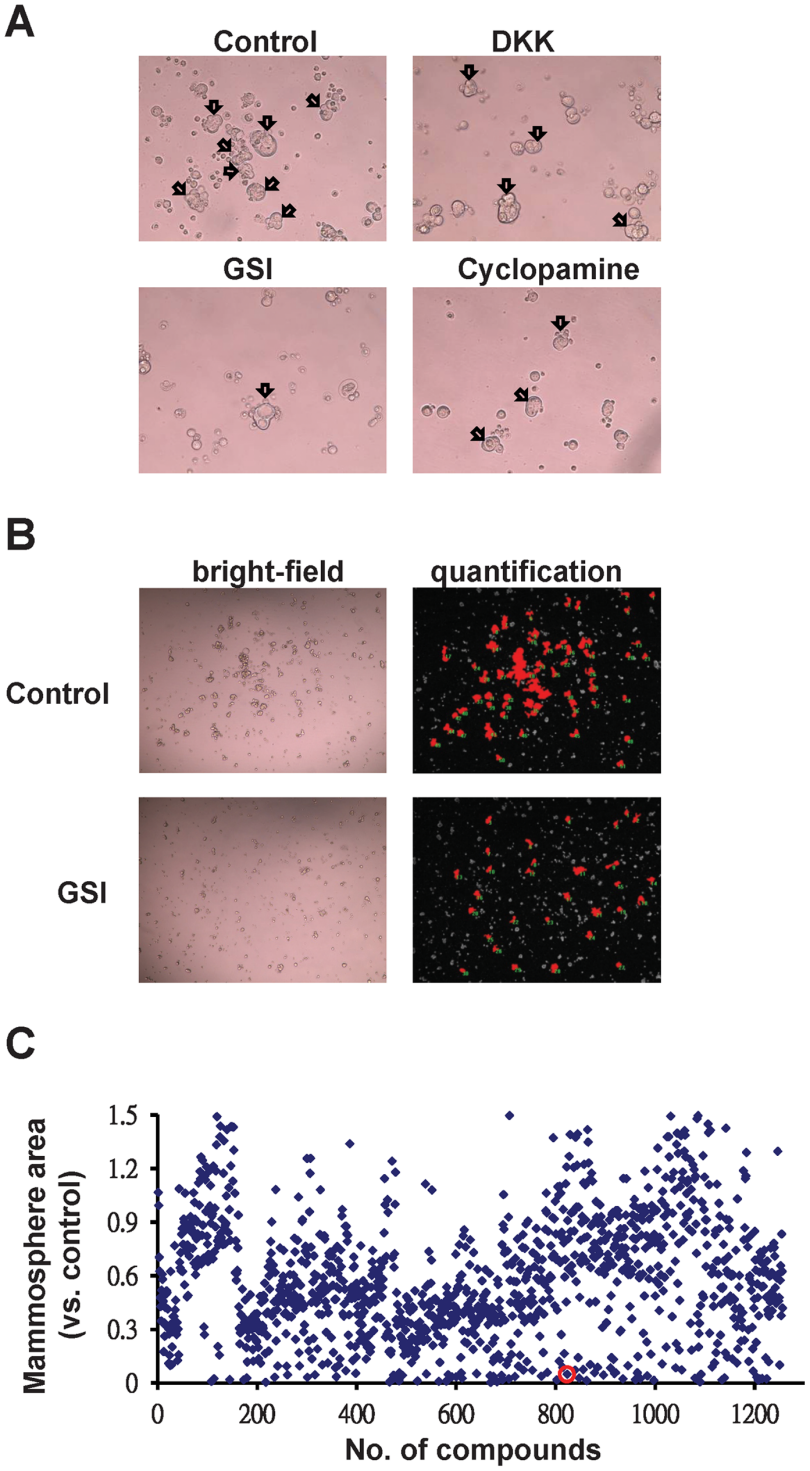
High throughput screening using SPS. (A) Wnt, Notch, and Hedgehog blockade inhibits MCF7 SPS formation. (magnification, 200×). (B) Image analysis of the area of tumorspheres after treatment with the Notch inhibitor GSI (30 µM) (magnification, 50×). (C) Chemical screening of compounds that inhibited SPS formation. Scatterplot of the 1,258 compounds of the LOPAC chemical library, screened against MCF7 SPS formation.

To test the feasibility of using these SPS for drug screening, inhibitors of stem cell signaling were used to test the inhibition of spheroid formation. We treated MCF7 SPS with stem cell signaling inhibitors, namely Dickkopf (DKK), r-secretase inhibitor (GSI), and cyclopamine, which are the inhibitor for Wnt, Notch, and Hh signaling, respectively, at a concentration of 2.1 mg/mL, 30 µM, and 30 µM, respectively, for 3 days. MCF7 SPS formation was significantly inhibited by these inhibitors (Figure 3A).These results suggest that the elaborate balance of these signaling pathways affects the self-renewal of MCF7 SPS. Therefore, screening compounds using this method may lead to the discovery of new therapeutic agents affecting breast cancer stem cells.

**Figure 4:**
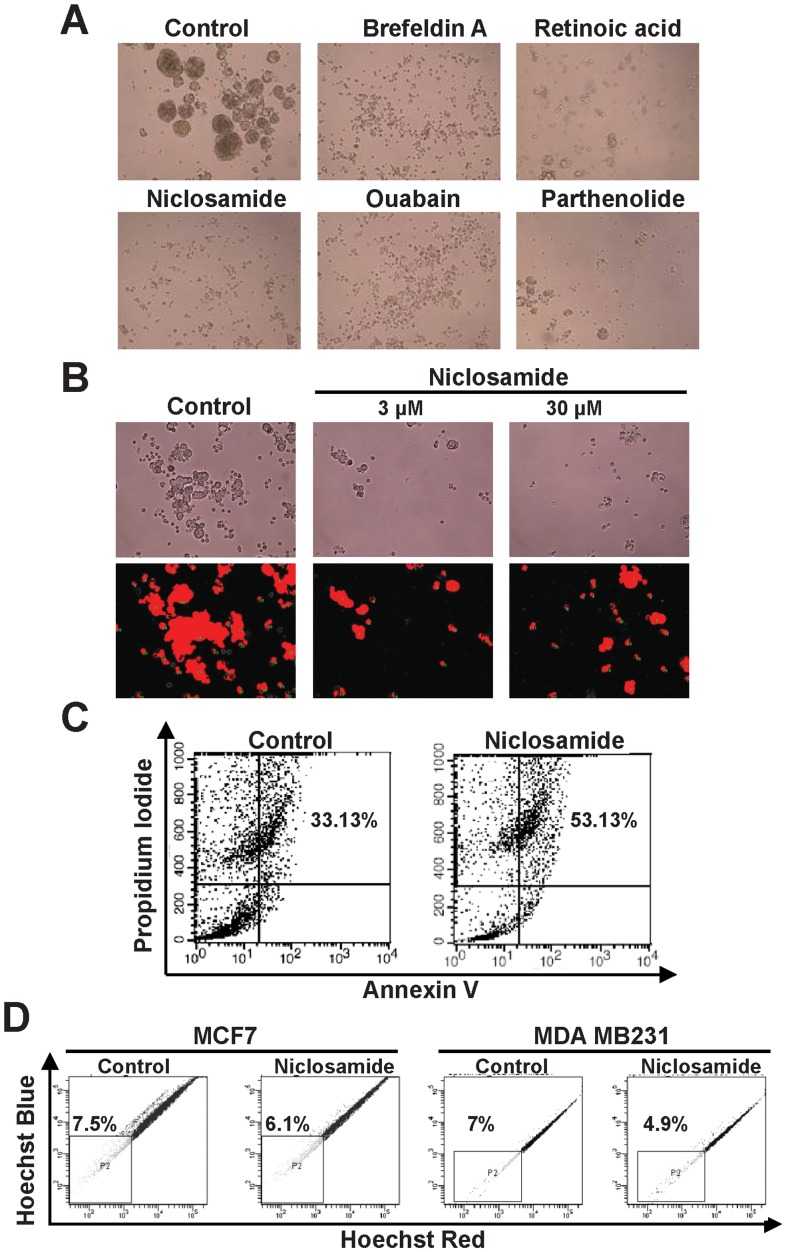
Examples of candidate compounds on drug screening. (A) The five selected compounds inhibited SPS formation (magnification, 200×). (B) Niclosamide inhibited MCF7 SPS formation. The SP of MCF7 was treated with 3 µM or 30 µM niclosamide for 48 h (magnification, 50×). Images of MCF7 SPS culture were analyzed using the Image-Pro Plus 6.0 software. (C) Niclosamide decreased the fraction of MCF7 SP. Treatment with niclosamide decreased the SP fraction in MCF7 and MDA-MB-231 cells. (D) MCF7 SPS were treated with niclosamide (3 µM) for 72 h. Apoptosis was evaluated using flow cytometry.

To perform a high-throughput drug screening, we established an image-based phenotypic assay to characterize SPS formation as a surrogate of drug effectiveness. MCF7 SP cells were incubated in 96-well plates to grow spheroids and MCF7 SPS were screened using the LOPAC chemical library, which includes 1,258 chemical compounds, to identify drugs that inhibit spheroid formation. The Notch signaling pathway inhibitor r-secretase (30 µmol/L) was used as a positive control and the areas of spheroids were analyzed using the Image-Pro Plus 6.0 software (Figure 3B). During the primary screening, about 307 compounds yielded a reduction of the area of MCF7 SPS at a concentration of 30 µM (Figure 3C). These 307 compounds were subjected to a second screening at a concentration of 3 µM, which narrowed down the candidate list to 39 compounds (Table 1). The inhibitory effects of five hits among these 39 compounds are shown in Figure 4A. Subsequently, we chose niclosamide as a potential lead for further studies. IC50 of niclosamide was first determinned by the standard dose response curve (Figure S1).To identify therapeutic strategies that target stem cells, treatment with niclosamide at a concentration of 3 µM decreased the area of spheroid by 60% of MCF7 SPS cells (Figure 4B) and treatment with niclosamide at a concentration of 5 µM decreased the SP fraction in MCF7 and MDA-MB-231 cells (Figure 4C). To determine the effect of niclosamide on SPS, we investigated the mechanism involved in this process. MCF7 SPS cells treated with niclosamide for 72 h were stained with annexin V/FITC and propidium iodide. Flow cytometry analysis revealed that niclosamide caused a considerable increase in apoptotic cell death, as assessed using annexin V/propidium iodide staining (Figure 4D). This result indicates that niclosamide causes MCF7 SPS death via apoptosis.

**Table 1:** Bioactive pharmacologic classes from LOPAC.

Class	Total agents	Active agents
Cytotoxic agents*	91	13
Biochemistry	43	2
Adenosine	53	1
Adrenoceptor	102	1
Hormone	31	1
Neurotransmitter	423	5
Intracellular Calcium	7	1
Ion pump and ion	72	4
Nitric Oxide	36	3
Phosphorylation	92	6
Tachykinin	5	2
	1258	39

* Includes Antibiotic, Apoptosis, Cell Cycle, Cyclic Nucleotides, Cytoskeleton and ECM, DNA Metabolism, Transcription etc.

### Effect of Niclosamide on Tumor Growth *in vivo*

According to the results of the *in vitro* (cell culture) analyses described above, we assessed further the therapeutic effects of niclosamide *in vivo*. MCF7 SPS (10^5^ cells) suspended in 100 µL of matrigel were injected subcutaneously into the fat pad of NOD/SCID mice. Subsequently, we treated mice with niclosamide (10 mg/kg) intraperitoneally 5 days per week for 8 weeks. The mice tolerated the therapy well and did not display apparent symptoms of cytotoxic effects of niclosamide at the testing doses. Tumor formation was assayed by palpation and mice were sacrificed 8 weeks after MCF7 SPS injection.

**Figure 5:**
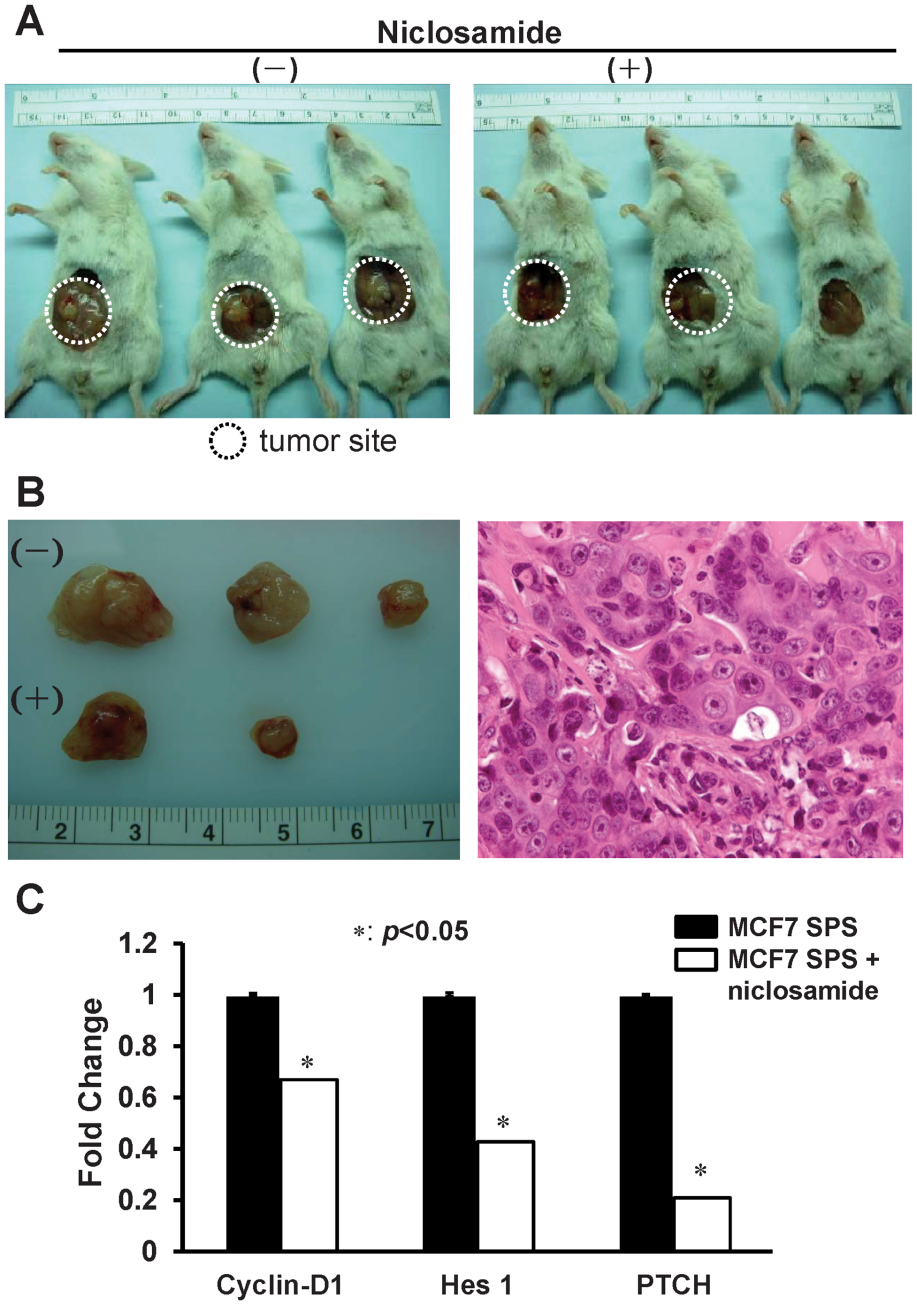
Effects of niclosamide on breast cancer SPS *in vivo*. (A) The growth of tumors from MCF7 SPS was perturbed in the niclosamide-treated group compared with the vehicle-treated group. (B) MCF7 SPS tumor-size of niclosamide treated NOD-SCID mice and histological analysis of tumors. (C) The mRNA expression of self-renewal signaling target genes was regulated by niclosamide (*P<0.05).

The results confirmed that niclosamide inhibits tumor growth and reduces tumor weight while the effect is on the borderline of statistical significance (Figure 5A,B and S2). The pathology confirmed the diagnosis of breast cancer (Figure 5B).

### Effect of Niclosamide on the Self-renewal Signaling Pathway

The Wnt, Notch, and Hedgehog pathways in normal stem cells and cancer stem cells have been the focus of intensive investigation. To investigate the mechanisms underlying the effect of niclosamide on stem signaling in breast cancer stem-like cells, the mRNA of representative target genes (*cyclin D1*, *Hes1*, and *PTCH*, which are the target genes for Wnt, Notch, and Hh signaling, respectively) were tested. Niclosamide treatment inhibited the expression of *cyclin D1*, *Hes1*, and *PTCH* by 33%, 57%, and 79%, respectively (Figure 5C).

## Discussion

The identification of drugs that specifically target cancer-initiating cells is a current and major challenge in breast cancer treatment. The present study developed a unique method for the enrichment of breast cancer stem cells and used these cells in a high-throughput drug screening using an image-based system. We successfully identify an old anthelmintic drug, niclosamide, which can target breast SPS subpopulations and inhibit tumor growth *in vivo.*

Chemical approaches using small molecules have provided a powerful method of interrogating biological processes, including cancer stem cells [23], [24]. Cell-based phenotypic screening assays of small molecules also offer a powerful chemical tool that can be used to identify effective drugs and study complex cellular processes. In recent years, several small molecules have been proven to be useful for targeting cancer progenitor cells and transformed cells, and various drug-screening platforms that were specifically designed to target cancer stem-like cells have successfully identified novel drugs, such as salinomycin, an antibiotic, and thioridazine, a dopamine antagonist [25], [26]. However, these hits are selected by the platform of either one of the subpopulation cancer cells. To better search for small molecules that affect cancer stem-like cells and can be used in breast cancer therapies, we combined side populations and spheroid formation assays with a cell-based assay to identify modulators that are used clinically and may remodel cancer stem-like cells and find applications in the treatment of breast cancer. Our recently published study, which was performed using a similar approach, also confirmed that niclosamide is an inhibitor of ovarian-cancer-initiating cells [27].

The mechanism via which niclosamide, a protonophoric anthelmintic drug, induces stem-like-cell-specific toxicity in breast cancer is interesting. It is an old drug that has been used to treat tapeworms in animals [28]. Niclosamide is known to uncouple mitochondrial oxidative phosphorylation during tapeworm killing. A screening of autophagy modulators revealed that niclosamide is a novel inhibitor of mTORC1 signaling [29]. A recent work also demonstrated that niclosamide induces the apoptosis of myelogenous leukemic cells via the inactivation of NF-kappaB and reactive oxygen species generation [30]. Niclosamide was also reported to inhibit Wnt signaling [31]–[33] in colon cancer cells. Our recent work demonstrated that niclosamide disrupts multiple metabolic pathways in ovarian-cancer-initiating cells [27]. The present study showed that niclosamide treatment resulted in the downregulation of target genes involved in the self-renewal of cancer stem-like cells and inhibited breast SPS. Our results support the potential of the small molecule niclosamide as a leading compound in breast cancer therapy.

In summary, we discovered an antiparasitic agent, niclosamide, via a high-throughput drug screening of cell-line-derived SPS. This drug inhibited the growth of breast cancer stem-like cell subpopulations *in vitro* and *in vivo*. As it is a clinically approved drug, the extension of niclosamide to clinical trials might be expedited, allowing the concept of targeting these cancer stem-like subpopulations in human breast cancer patients to be assessed in the near future.

## Supporting Information

Figure S1 **The dose response curves of MCF7 and MDA-MB- 231 breast cancer cells treated with niclosamide.**
(TIF)

Figure S2 **Tumors developed from MCF7 SPS with niclosamide treatment or vehicle control were weighted (** ***P*** **= 0.09).**
(TIF)
