# Supplementary figures and images for: Drug Screening Identifies Niclosamide as an Inhibitor of Breast Cancer Stem-Like Cells
Source: PLoS One. 2013 Sep 18;8(9):e74538. doi: 10.1371/journal.pone.0074538 (PMC3776833; doi:10.1371/journal.pone.0074538)

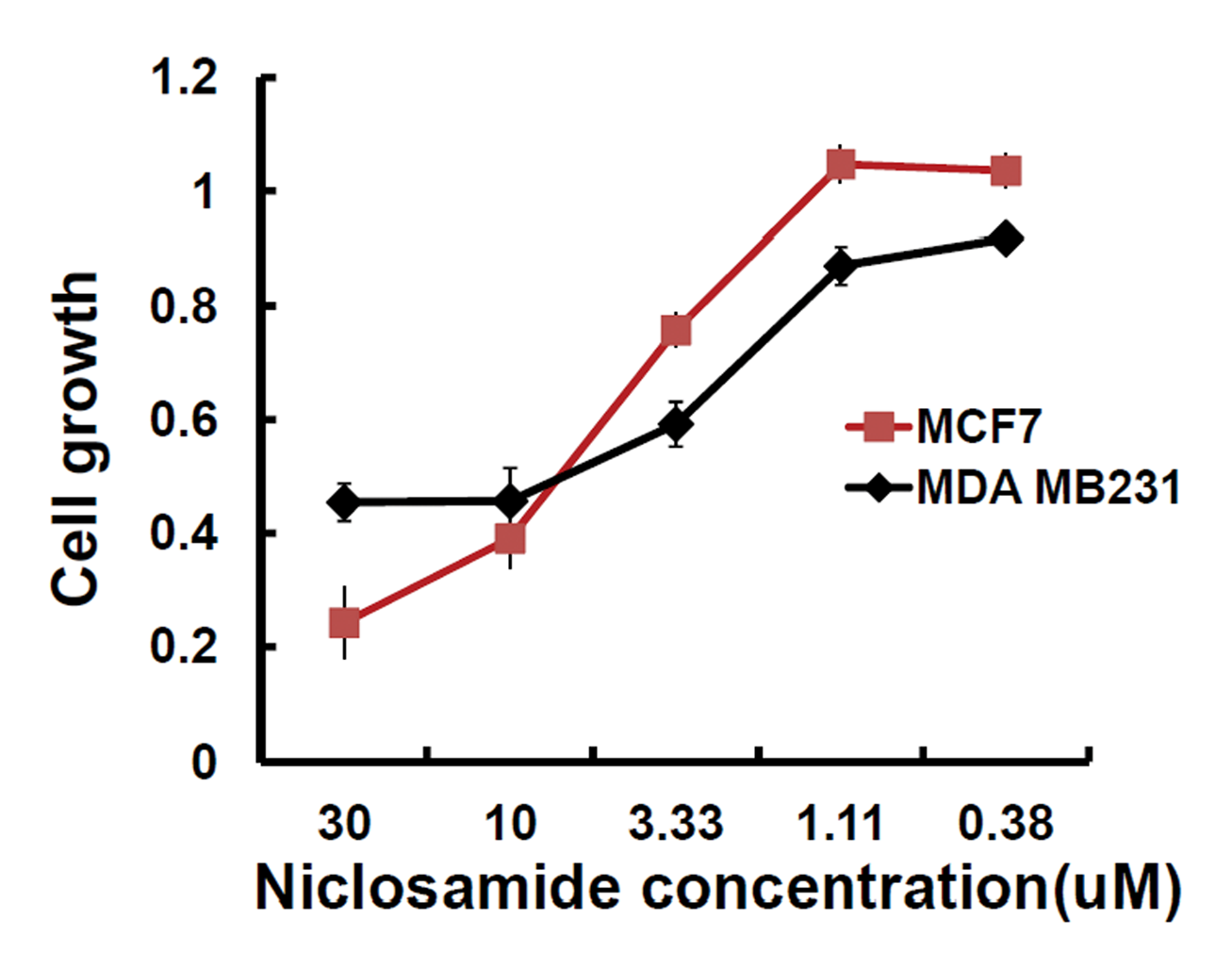

Supplement: Figure S1 — The dose response curves of MCF7 and MDA-MB- 231 breast cancer cells treated with niclosamide. (TIF) [file pone.0074538.s001.tif]

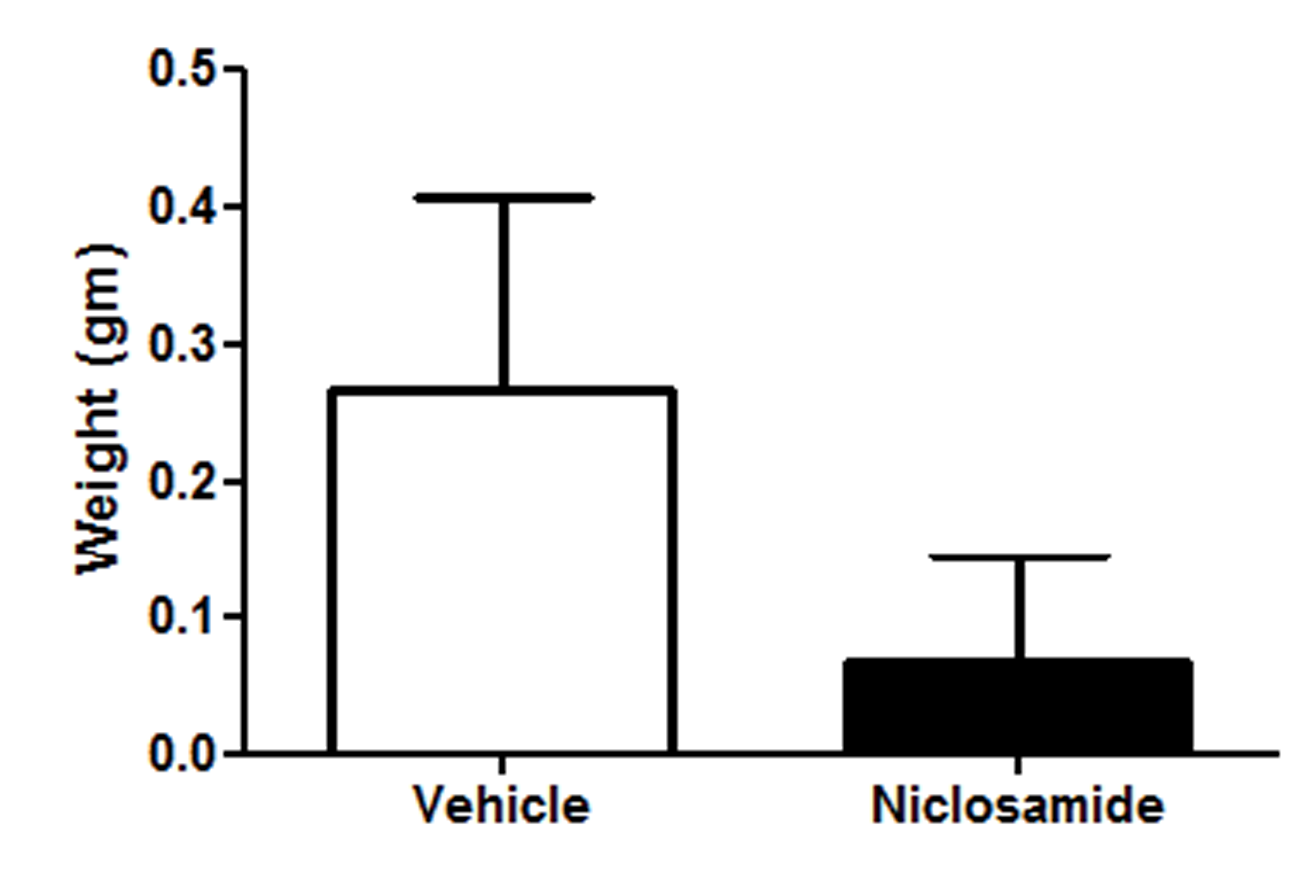

Supplement: Figure S2 — Tumors developed from MCF7 SPS with niclosamide treatment or vehicle control were weighted ( P = 0.09). (TIF) [file pone.0074538.s002.tif]
